# Mitigation of COVID-19 at the 2021 National Collegiate Athletic Association Men’s Basketball Tournament

**DOI:** 10.1186/s12889-022-14547-1

**Published:** 2022-11-10

**Authors:** Brian E. Dixon, William F. Fadel, Thomas J. Duszynski, Virgina A. Caine, Joeseph F. Meyer, Michele Saysana

**Affiliations:** 1 Fairbanks School of Public Health, Indiana University, 1050 Wishard Blvd. Floors 5 and 6, Indianapolis, IN 46202 USA; 2 Center for Biomedical Informatics, Regenstrief Institute, Indianapolis, IN USA; 3 Marion County Public Health Department, Indianapolis, IN USA; 4 Indiana University School of Medicine, Indianapolis, IN USA; 5 Indiana University Health, Indianapolis, IN USA

**Keywords:** COVID-19 pandemic, SARS-CoV-2 virus, Public Health Surveillance, Communicable disease control, Infectious disease transmission, Basketball, Sports, Athletes

## Abstract

**Background:**

Data are lacking regarding the risk of viral SARS-CoV-2 transmission during a large indoor sporting event involving fans utilizing a controlled environment. We sought to describe case characteristics, mitigation protocols used, variants detected, and secondary infections detected during the 2021 National Collegiate Athletic Association (NCAA) Men’s Basketball Tournament involving collegiate athletes from across the U.S.

**Methods:**

This retrospective cohort study used data collected from March 16 to April 3, 2021, as part of a closed environment which required daily reverse transcription-polymerase chain reaction (RT-PCR) testing, social distancing, universal masking, and limited contact between tiers of participants. Nearly 3000 players, staff, and vendors participated in indoor, unmasked activities that involved direct exposure between cases and noninfected individuals. The main outcome of interest was transmission of SARS-CoV-2 virus, as measured by the number of new infections and variant(s) detected among positive cases. Secondary infections were identified through contact tracing by public health officials.

**Results:**

Out of 2660 participants, 15 individuals (0.56%) screened positive for SARS-CoV-2. Four cases involved players or officials, and all cases were detected before any individual played in or officiated a game. Secondary transmissions all occurred outside the controlled environment. Among those disqualified from the tournament (4 cases; 26.7%), all individuals tested positive for the Iota variant (B.1.526). All other cases involved the Alpha variant (B.1.1.7). Nearly all teams (*N* = 58; 85.3%) reported that some individuals had received at least one dose of a vaccine. Overall, 17.9% of participants either had at least one dose of the vaccine or possessed documented infection within 90 days of the tournament.

**Conclusion:**

In this retrospective cohort study of the 2021 NCAA Men’s Basketball Tournament closed environment, only a few cases were detected, and they were discovered in advance of potential exposure. These findings support the U.S. Centers for Disease Control and Prevention (CDC) guidelines for large indoor sporting events during the COVID-19 pandemic.

## Background

Mass gatherings, including indoor sporting events, are associated with the transmission of SARS-CoV-2 [1], the virus that causes coronavirus disease 2019 (COVID-19). Not only are attendees of these events exposed to others with active infection, especially given that many individuals are asymptomatic [2], but individuals infected at large events often cause secondary infections in the community, dubbing mass gatherings as ‘super-spreader’ events. Because of their potential impact on community spread, public health guidelines and policies across the United States restricted or prohibited mass gatherings for most of 2020, resulting in cancellations of many large sporting events [3]. Cancelled events for 2020 included the National Collegiate Athletic Association (NCAA) Men’s and Women’s college basketball tournaments, known as March Madness, as well as most conference basketball tournaments and the remaining winter and spring NCAA tournaments [4].

When large sporting events and tournaments resumed, event organizers followed various mitigation protocols based on recommendations from the Centers for Disease Control and Prevention (CDC) as well as local public health departments. For example, the National Basketball Association (NBA) implemented a controlled campus environment in which all games were played in a single sports complex without fans and where players were confined to a limited set of facilities for the duration of the multi-day event [5]. Major League Baseball did not use a controlled environment. Although fans were not allowed in the stadiums, teams still travelled to and played games in multiple cities while following mitigation protocols involving testing and quarantining [6]. Beyond models that estimate secondary infections [7], there exists no evidence from large indoor sporting events in which fans were allowed. Moreover, there is little evidence on the impact of prior infections and vaccination on risk of infection due to large indoor sporting events where fans are present.

In this analysis, we examine the impact of SARS-CoV-2 infection mitigation protocols implemented by the NCAA for its 2021 Men’s Basketball Tournament held in Indianapolis, Indiana in late March and early April 2021. We outline the mitigation protocols in place to keep involved athletes, coaches, and others safe, detail positive cases and clusters identified during the tournament, examine the influence of genetic variants of SARS-CoV-2, and explore the impact of prior infection and vaccination on risk for secondary infection.

## Methods

The NCAA hosted its 2021 March Madness tournament using a layered set of mitigation strategies. Informed by CDC guidelines, recommendations from its medical advisory board, local public health agencies, and host venues, the NCAA developed and implemented a comprehensive plan involving a controlled environment, regular screenings, social distancing, and masks for everyone involved in the tournament. The controlled environment required that players, coaches, NCAA officials, and support staff remain in regulated, designated areas (including living areas, transportation, and basketball venues) that adhered to mitigation protocols including rigorous cleaning regimens. Teams could not leave the environment once they arrived at the tournament, and movement into and out of the environment was dictated by the mitigation protocols, especially multiple negative tests prior to entry.

Protocols were developed in late 2020 with final sign off from the Marion County Public Health Department, the jurisdiction responsible for the main site of the tournament, on February 1, 2021, when final capacity (25%) for fans was determined based on local epidemiology of SARS-CoV-2. This retrospective cohort study was approved by the Indiana University (IU) institutional review board and followed Strengthening the Reporting of Observational Studies in Epidemiology guidelines for cohort studies.

### Testing, social distancing, and masking

The NCAA categorized individuals into tiers based on its COVID-19 Guidance on Multiple Teams in the Same Location [8], derived from DiFiori et al. [9] Tier 1 involved individuals for whom physical distancing and face coverings were not used, including athletes, coaches, medical staff, trainers, and officials. Individuals in Tier 2 came into close contact with Tier 1 individuals but generally maintained physical distance and used face coverings. This included bus drivers, NCAA administrative staff, and event security. Tier 3 individuals provided event services, but they did not come into close contact with Tier 1 individuals. These individuals included housekeeping, catering, and the media. Tier 4 represented fans admitted to the venue but restricted from interacting with Tier 1 individuals. For example, the parents of players were not allowed to have any in-person contact during the tournament.

Table 1 details the mitigation strategies observed during the tournament. Tier 1 individuals were required to test negative for seven consecutive days prior to arrival in Indianapolis. These individuals were isolated upon arrival until they screened negative for SARS-CoV-2 virus twice. Tier 2 individuals were tested upon arrival and placed into quarantine until a single negative result. All individuals were expected to observe universal masking and social distancing throughout the tournament.

**Table 1:** SARS-CoV-2 infection mitigation strategies implemented for the NCAA Men’s Basketball Tournament held March 18 – April 2, 2021 in Indianapolis, Indiana, USA

	Testing	Social Distancing and Masks
Tier 1	• Pre-Arrival: 7 consecutive daily tests prior to arrival, one of which must be RT-PCR • During Tournament: Daily testing using RT-PCR nasal swab, including day of arrival	• Placed into quarantine upon arrival. Remain until two consecutive negative tests • Each team had its own floor of the hotel to limit interaction between teams • Observe universal masking and physical distancing unless eating, practicing, or competing • Travel, meetings and meals had seating charts to aid contact tracing • Players wore Kinexon© wristbands to monitor interactions with other players during practices and games
Tier 2	• No pre-arrival requirement • Tested upon arrival using RT-PCR nasal swab • Tier 2 tested 2 × per week • Positive test resulted in individual leaving the controlled environment	• Placed into quarantine upon arrival until negative test is confirmed • Observe universal masking and physical distancing • Limited interaction with Tier 1 individuals
Tier 3	• No pre-arrival requirement • Tested upon arrival using RT-PCR nasal swab • Tier 3 tested 2 × per week • Positive test resulted in individual leaving the controlled environment	• Observe universal masking and physical distancing • Ad hoc interactions with Tier 1 individuals
Tier 4	• No requirements	• Observe universal masking and physical distancing • Restricted from interactions with Tier 1 individuals

All Tier 1 individuals were housed in Marion County, Indiana, within the controlled environment. Tournament buses transported Tier 1 individuals to venues. Tier 1 individuals were not allowed to enter any facilities outside tournament venues, with buses returning to designated hotels in Indianapolis after competition. Each team was housed on a separate floor of a designated hotel, and workout and practice venues were staggered and cleaned between uses, limiting interaction and exposure when not in competition. Players were socially distanced when in transport. Within teams, individuals could interact during meals, practices, workouts, games, and on their hotel floor. When not practicing or competing, individuals observed universal masking.

Indiana University Health, the state’s largest health system with a state-of-the-art clinical laboratory facility and academic medical center in downtown Indianapolis, was responsible for screening and testing individuals in Tiers 1 and 2 daily. All Tier 1 individuals received a daily self-administered, observed anterior nasopharyngeal (NP) swab. Specimens were pooled for analysis in small (*N* = 5) batches and run 6–8 h after collection. If any specimen returned positive, then all samples in the pool would be tested individually. If the confirmatory test was positive, the individual was presumed positive and placed into isolation. If the individual was asymptomatic, a second test was performed to confirm active infection. Should the second test come back negative, then a third test was performed. The individual remained in isolation until both second and third tests could be analyzed to confirm negative status. Individuals confirmed to be positive were excluded from the rest of the tournament and sent home for the remainder of their quarantine period.

Laboratory diagnostics were performed using assays with emergency use authorization to detect viral RNA via RT-PCR by IU Health on either the Roche cobas® 8800 System or Roche cobas® Liat® PCR System. Anterior NP swabs were placed in single tubes before being assayed. Most tests were conducted on the Roche cobas® 8800 System. If a screening test was positive, a second test was performed on the Roche cobas® Liat® PCR System. The cobas® SARS-CoV-2 Test performed on the cobas® 8800 System is a single-well dual target assay, which includes both specific detection of SARS-CoV-2 and pan-sarbecovirus detection for the sarbecovirus subgenus family that includes SARS-CoV-2. The test detects the genetic signature (RNA) of the SARS-CoV-2 virus in nasal, nasopharyngeal, and oropharyngeal swab samples.

Individuals at least two weeks post-infection and within 90 days of the first known date of infection were excluded from the daily testing regimen. Any individual with a documented positive SARS-CoV-2 infection after December 5, 2020, was excused from pre-arrival testing requirements.

### Data

Our data include longitudinal laboratory tests collected before and during the NCAA tournament for all participating players, staff, and vendors (e.g., bus drivers). History of prior infection and vaccination records were documented using team attestations completed by university health officials, including team doctors. These records were linked to laboratory data using name and date of birth. Out of 2,738 individuals tested on site, 2,660 (97.2%) were matched to attestation records. Individuals were grouped into their respective Tiers based on designations from the NCAA. Data further included the results of genotyping performed by IU Health for each positive PCR result.

### Statistical analysis

Descriptive statistics (counts and proportions) were calculated for (1) individuals partially vaccinated (1 dose of an mRNA vaccine), (2) individuals fully vaccinated (2 doses of an mRNA vaccine or 1 dose of the Ad26.COV2.S vaccine), and (3) individuals who reported an active infection within 90 days (December 8, 2020 or later) of the start of the Tournament. Analyses were conducted using R version 4.0 (R Core Team).

## Results

**Fig. 1:**
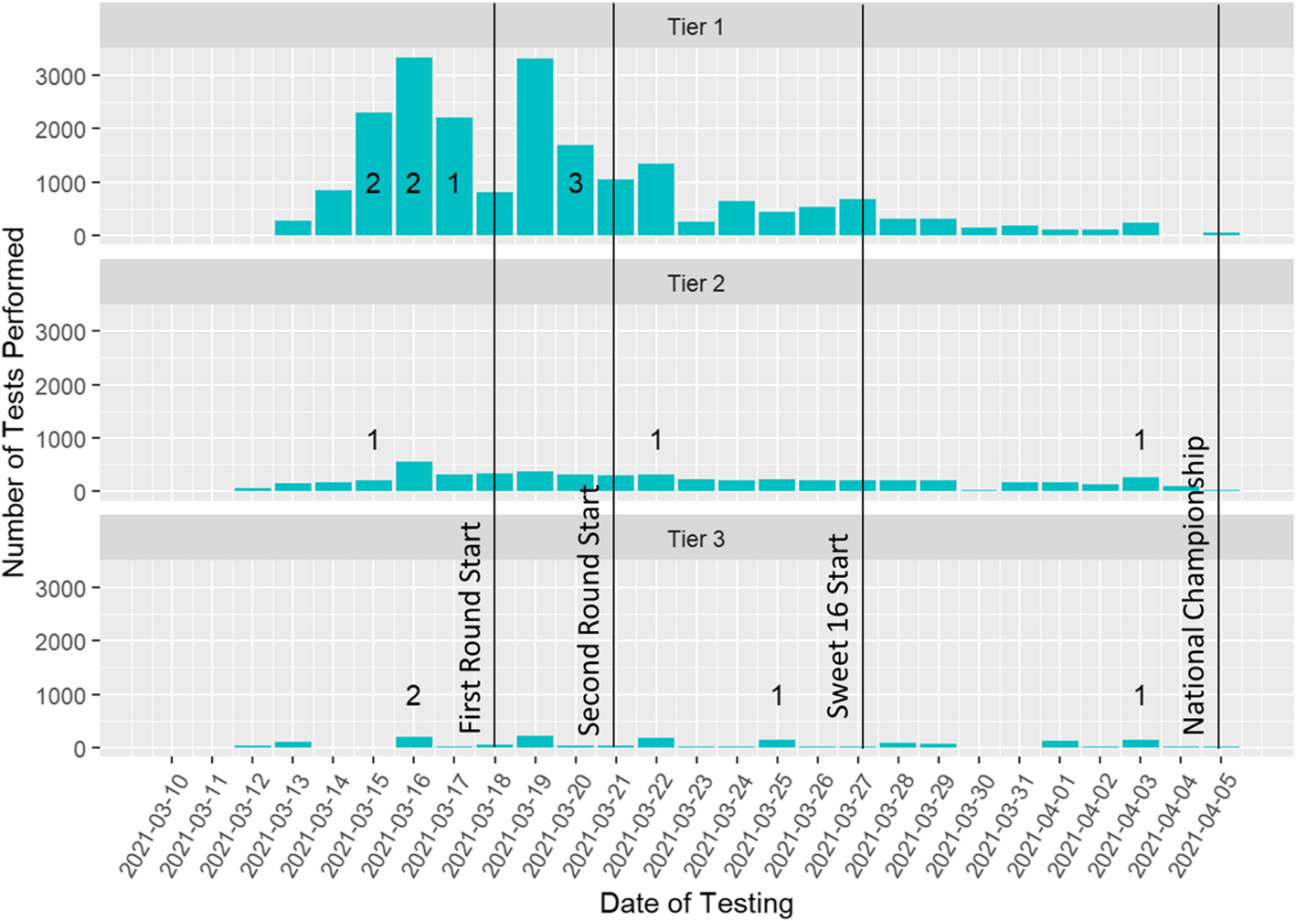
Volume of SARS-CoV-2 laboratory testing before and during the 2021 NCAA Men’s Basketball Tournament by date, stratified by participant tier. Numbers indicate positive results. Milestones in the tournament are also noted, such as when the first round of games began

A total of 28,311 tests were conducted over 26 days for individuals involved in the tournament. Out of the matched cohort with 2,660 people, a total of 15 individuals (0.56%) screened positive. Figure 1 summarizes laboratory testing volumes by day of the tournament along with the date of positive cases, stratified by participant tier. Tournament milestones, such as the start of the first round, are marked on the figure. The volume of testing declined over time as teams were eliminated from the tournament, resulting in the eliminated teams leaving Indianapolis.

### Tier 1 cases

Eight cases (53.3%) occurred among Tier 1 participants, and all cases were detected before any individual played in or officiated a game. Prior to the first round of play, three players, one school administrator, and one official tested positive. Two of the players were detected while in quarantine upon arrival in Indianapolis, so they were sent home and found to have no close contacts. The school administrator returned home before their team played in the first round, and their contact with players was limited. The official tested positive before officiating any games. Furthermore, contact tracing linked the official to five individuals with whom he had close contact outside of the controlled environment. These individuals, including other officials, were excluded from the tournament.

The third player was detected one day after quarantine ended, which enabled him to eat and practice with his team. Two days later, three additional individuals associated with the third player’s team also tested positive. Because multiple cases were detected within the same team, the team was disqualified for tournament play and sent home before their first-round game, limiting exposure to other Tier 1 individuals. The three subsequent cases are not suspected to be caused by the first player given their timing. Contact tracing revealed the team was most likely exposed while playing in a conference tournament prior to arrival in Indianapolis. Also linked to the conference tournament was the official who tested positive. Exposure likely occurred in a hotel where multiple teams and individuals co-mingled in common spaces and elevators, which was prohibited in Indianapolis.

### Tier 2 cases

Three cases (20%) occurred among Tier 2 individuals, and no Tier 2 individuals were linked to any other cases. The individual who tested positive at the start of the tournament was a digital media company employee. Moreover, they originally tested positive on March 3, 2021; therefore, retesting was not required according to NCAA protocols. They were asked to quarantine until March 17, 2021, and then they could come back to work inside the controlled environment. The second case involved a bus driver who did not socially distance when in the community. Contact tracing revealed that this driver engaged in eating and drinking with other bus drivers, although none of those individuals tested positive. The third case involved an NCAA administrative staff person who did not interact with teams.

### Tier 3 cases

There were four cases (26.7%) among Tier 3 individuals. Two cases identified on March 16 and one identified on March 25, were travel company employees who provided logistical support. These individuals attended a bourbon tasting event in the community prior to the start of the tournament where they were in close contact with one another for an extended period of time. The third individual, a close contact of the other two cases, tested positive on Day 10 of quarantine. The final case involved a NCAA Men’s Basketball Committee member who travelled to Indianapolis for the final four games and tested positive before attending any games.

### Genetic sequencing

**Fig. 2:**
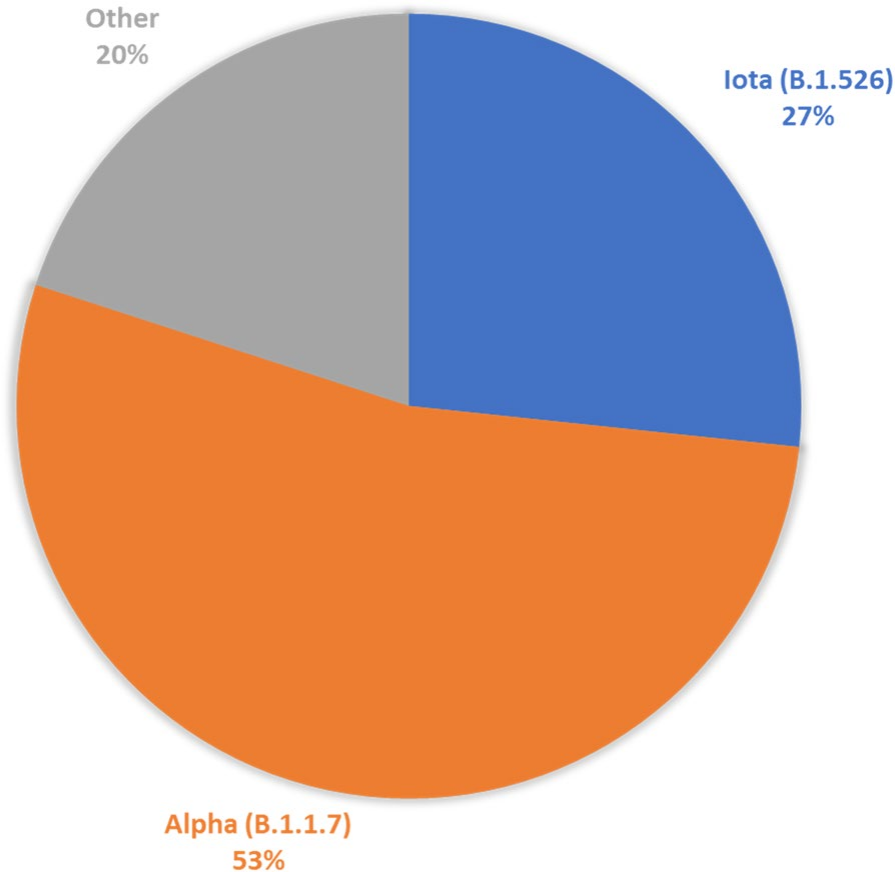
Genetic sequencing for positive COVID-19 cases detected during the 2021 NCAA Men’s Basketball Tournament, stratified by variant of the SARS-CoV-2 virus

Sequencing results are summarized in Fig. 2. All individuals associated with the team disqualified from the tournament (4 cases; 26.7%) tested positive for the Iota variant (B.1.526), a lineage predominantly circulating in New York starting in November 2020 monitored by CDC as a variant of interest [10]. All other sequenced cases (8 cases; 53.3%) involved the Alpha variant (B.1.1.7), a lineage predominantly from the United Kingdom first detected in the U.S. at the end of 2020 and classified during the tournament by CDC as a variant of concern [11]. Three cases could not be sequenced due to relatively low presence of the virus in the specimens.

At the start of the tournament, the Alpha and Iota variants accounted for 21.3% and 5.4% of cases in the U.S., respectively. By the end of the tournament, Alpha and Iota variants accounted for 51.9% and 11.5% of cases, respectively. These estimates come from GISAID [12], a global initiative that maintains a repository of virus sequence data. Neither variant is associated with more severe disease. The Alpha variant (B.1.1.7) is associated with more efficient transmission than ancestral strains (e.g., those from 2020) and was predominant in the U.S. by the end of March 2021 {McCormick, 2022 #4285}.

### Immunity against SARS-CoV-2 virus among participants in tiers 1–3

Just under 1-in-5 participants in Tier 1 (18.9%) had some level of immunity against SARS-CoV-2 at the start of the tournament. As summarized in Table 2, about 1-in-10 individuals across tiers were fully vaccinated ahead of the tournament. Immunity levels were highest among Tier 1 participants and lowest among Tier 3 participants. Recent infection was highest among Tier 2 participants and lowest among Tier 3 participants.

**Table 2:** Proportion of participants in Tiers 1–3 with partial vaccination, full vaccination, or recent infection due to SARS-CoV-2

Tier	Individuals in Tier N	Individuals Partially Vaccinated N (%)	Individuals Fully Vaccinated N (%)	Individuals with SARS-CoV-2 Infection within 90 Days of Tournament N (%)	Individuals with Prior Infection or at Least One Dose COVID-19 Vaccine N (%)
Tier 1	2064	94 (4.6)	242 (11.7)	62 (3.0)	391 (18.9)
Tier 2	294	5 (1.7)	36 (12.2)	11 (3.7)	51 (17.4)
Tier 3	302	1 (0.3)	29 (9.6)	4 (1.2)	34 (11.3)

The mean vaccination (partial or full) rate among participating teams was 15.7% (Median 9.2%; SD 19.3%; Range 0%-97%). Full information reported from the 68 teams (anonymized) is summarized in Table 3. Most teams (N = 58; 85.3%) reported that some individuals had received at least one dose of a vaccine. Nineteen teams (27.9%) had full vaccination rates higher than 13.0%, which was the U.S. mean rate at the start of the NCAA tournament. Thirteen teams (19.1%) reported that no one on the team was fully vaccinated, and 12 teams (17.6%) reported that no one possessed any dose of the vaccine. Most vaccinations were reported among coaching and training staff, who likely met age-based criteria used by states to rollout vaccinations. However, one team had every player and most coaches vaccinated.

**Table 3:** Proportion of Tier 1 participants reported to have partial vaccination, full vaccination, or recent infection due to SARS-CoV-2 according to team attestations, stratified by team

Team Name (Anonymized)	Partially Vaccinated (%)	Fully Vaccinated (%)	Full or Partially Vaccinated (%)	Previously Infected (%)	Prior Infection and/or Fully or Partially Vaccinated (%)
Team 1	0.0	97.0	97.0	3.0	97.0
Team 2	76.47	17.65	94.12	2.94	94.12
Team 3	0.0	14.71	14.71	47.06	61.76
Team 4	0.0	50	50	0.0	50
Team 5	28.12	15.62	43.75	3.12	46.88
Team 6	19.23	26.92	46.15	0.0	46.15
Team 7	5.56	38.89	44.44	0.0	44.44
Team 8	23.53	5.88	29.41	14.71	41.18
Team 9	0.0	39.39	39.39	3.03	39.39
Team 10	9.68	29.03	38.71	0.0	38.71
Team 11	29.03	9.68	38.71	0.0	38.71
Team 12	20.59	11.76	32.35	2.94	35.29
Team 13	25.81	6.45	32.26	0.0	32.26
Team 14	3.03	27.27	33.33	0.0	30.3
Team 15	6.45	22.58	29.03	0.0	29.03
Team 16	8	20	28	0.0	28
Team 17	0.0	9.09	9.09	18.18	27.27
Team 18	2.94	8.82	11.76	17.65	26.47
Team 19	0.0	23.33	23.33	0.0	23.33
Team 20	0.0	0.0	0.0	20.69	20.69
Team 21	9.38	9.38	18.75	0.0	18.75
Team 22	0.0	14.81	14.81	3.7	18.52
Team 23	0.0	18.52	18.52	0.0	18.52
Team 24	0.0	16.67	16.67	0.0	16.67
Team 25	0.0	9.09	9.09	6.06	15.15
Team 26	0.0	11.11	14.81	3.7	14.81
Team 27	0.0	5.88	5.88	8.82	14.71
Team 28	0.0	14.71	14.71	0.0	14.71
Team 29	0.0	13.79	13.79	0.0	13.79
Team 30	0.0	6.45	6.45	6.45	12.9
Team 31	0.0	12.9	12.9	0.0	12.9
Team 32	3.03	9.09	12.12	0.0	12.12
Team 33	0.0	12	12	0.0	12
Team 34	2.94	8.82	11.76	0.0	11.76
Team 35	0.0	3.57	3.57	7.14	10.71
Team 36	3.57	7.14	10.71	0.0	10.71
Team 37	7.14	3.57	10.71	0.0	10.71
Team 38	5.26	5.26	10.53	0.0	10.53
Team 39	9.68	0.0	9.68	0.0	9.68
Team 40	0.0	9.38	9.38	3.12	9.38
Team 41	0.0	6.25	6.25	3.12	9.38
Team 42	0.0	5.88	5.88	2.94	8.82
Team 43	0.0	8.82	8.82	0.0	8.82
Team 44	0.0	8.33	8.33	0.0	8.33
Team 45	0.0	8.33	8.33	0.0	8.33
Team 46	0.0	3.85	3.85	3.85	7.69
Team 47	0.0	0.0	0.0	7.41	7.41
Team 48	0.0	7.41	7.41	0.0	7.41
Team 49	0.0	7.14	7.14	0.0	7.14
Team 50	0.0	6.9	6.9	0.0	6.9
Team 51	0.0	6.45	6.45	0.0	6.45
Team 52	0.0	5.88	5.88	0.0	5.88
Team 53	0.0	4.55	4.55	0.0	4.55
Team 54	0.0	4	4	0.0	4
Team 55	0.0	3.57	3.57	0.0	3.57
Team 56	0.0	3.33	3.33	3.33	3.33
Team 57	0.0	3.12	3.12	0.0	3.12
Team 58	0.0	3.03	3.03	0.0	3.03
Team 59	0.0	0.0	0.0	0.0	0.0
Team 60	0.0	0.0	0.0	0.0	0.0
Team 61	0.0	0.0	0.0	0.0	0.0
Team 62	0.0	0.0	0.0	0.0	0.0
Team 63	0.0	0.0	0.0	0.0	0.0
Team 64	0.0	0.0	0.0	0.0	0.0
Team 65	0.0	0.0	0.0	0.0	0.0
Team 66	0.0	0.0	0.0	0.0	0.0
Team 67	0.0	0.0	0.0	0.0	0.0
Team 68	0.0	0.0	0.0	0.0	0.0

Nearly one third (N = 22; 32.4%) of teams reported that at least one individual had a prior infection within 90 days of the tournament. The mean prior infection rate of 2.8% (Median 0.0%; SD 7.1%; Range 0%-47.1%) was much lower than the full vaccination rate. Five teams (7.4%) reported that somewhere between 15 and 47% of their Tier 1 individuals were infected recently. Infections were clustered in early February, about 6 weeks before the NCAA Tournament.

### Tier 4 infection rates

**Fig. 3:**
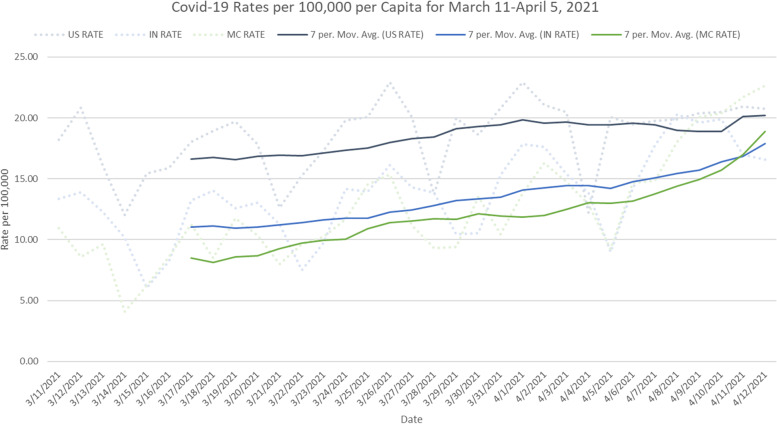
COVID-19 infection rate per 100,000 population for Marion County, Indiana (MC), the State of Indiana (IN), and the United States (US) between March 11 and April 12, 2021. Dotted lines represent the daily infection rates per 100,000 population. Solid lines represent 7-day moving average for infection rate per 100,000 population

Infection rates per 100,000 population for Indianapolis, the State of Indiana, and the United States during the March Madness timeframe are summarized in Fig. 3. The national rate rose gradually from just above 16 per 100,000 to 20 per 100,000 just prior to the Final Four. Then the U.S. rate remained flat through one week after the tournament. The rate climbed for the State of Indiana from just above 10 per 100,000 to 18 per 100,000 one week after the tournament ended. The rate for Indianapolis, which encompasses nearly all of Marion County, Indiana, also climbed from just below 10 per 100,000 to nearly 19 per 100,000 one week after the tournament ended. The steepest increases occurred just after the end of the tournament.

## Discussion

The controlled environment with robust mitigation strategies used by NCAA and its host city provides a model for conducting large sporting events in the middle of a global pandemic for an emerging infectious disease. Universal masking and distancing combined with limited interaction and daily testing within a controlled environment reduced exposure while enabling maximal sensitivity in the detection of positive cases. Just 0.5% of individuals in the controlled environment tested positive during the tournament when nationally the CDC reported a positivity rate of 4.1%. Moreover, protocols to limit non-play interaction further protected others involved with the event.

All cases among Tier 1 individuals directly involved in tournament play were identified upon arrival, prior to first-round games. Frequent testing prior to arrival in Indianapolis also identified individuals before entry into the controlled environment. Secondary transmission occurred outside the controlled environment, either in the community at a restaurant or private venue, or prior to arrival in Indianapolis. This is in stark contrast to open sporting events like major league baseball, in which players come and go from facilities. During a single three-game series in Philadelphia involving 146 individuals, 20 players (Tier 1) tested positive and exposures were linked to non-play activities on site [6]. The NCAA tournament saw fewer than 20 individuals test positive out of 2,849 individuals involved in the controlled environment. This rate is similar to the one observed by the NBA [5], which also employed a controlled environment.

An additional factor that likely contributed to the safety of the athletes and staff, above and beyond the controlled environment and protocols, was the fact roughly 1-in-5 individuals possessed some level of immunity to SARS-CoV-2. Several teams had vaccination rates higher than the U.S. rate at the time of the tournament (13.0%). Moreover, several individuals (2.8%) reported a recent infection. Although reinfections have been shown to occur in individuals with infection-induced as well as vaccine-induced immunity [13–15], prior studies suggest that immunity from natural infection can last up to 8 months [16]. Bozio et al. [17] found that vaccination provides 5-times protection compared with natural infection. Antibodies in 20% of participants likely helped protect some athletes and staff.

The impact of the tournament to Indianapolis, Indiana, and the Nation was likely negligent at best. Despite the observed rise in the infection rate per 100,000 population one week after the tournament ended, similar to O'Donoghue [18], concluding community spread is attributable to the NCAA tournament would be an ecological fallacy. The tournament concluded during western orthodox Easter weekend, when many large churches held indoor gatherings and many families likely had close contact indoors with extended relatives. Moreover, the final weekend occurred at the end of Passover, a week-long religious celebration. The study by Vest et al. [19] suggests around 75% of spectators were correctly masked inside NCAA tournament venues. Given a very low rate of infections within the environment and limited secondary transmissions linked to active cases, it is unlikely the NCAA tournament contributed meaningfully to community spread.

### Strengths and limitations

The primary strengths of this study include the availability of detailed epidemiologic and daily quantitative results in a large, closed cohort with measured engagement in regular contact without masking and physical distancing among a subset of individuals. Importantly, this study was able to control for sources of virus exposure because of the nature of the Indianapolis campus and the multiple tests prior to travel. Further, to our knowledge, this study is the first to include observations from repeated unmasked exposures between recovered SARS-CoV-2 positive individuals with prolonged viral shedding, vaccinated individuals, and presumed SARS-CoV-2-naïve individuals. We did not observe evidence of viral transmission among these individuals within the controlled environment. This finding provides additional evidence that individuals are unlikely to have replication-competent virus as a recovered individual. Like the NBA study [5], this study reports findings among ambulatory immunocompetent individuals who were being tested daily within a closed cohort.

The limitations of this retrospective cohort study include limited ability to confirm whether any secondary infections occurred once infected individuals returned home. Although we contacted local health authorities outside Indiana, few provided any response. Second, data on vaccination and prior infection relied upon attestation by each team’s medical doctor. Vaccination cards and prior test results were not confirmed, potentially underreporting individuals with at least one dose of a vaccine or asymptomatic infection. Finally, data reflect infections with the predominant strains during the study period and may not be applicable to other variant strains of SARS-CoV-2.

## Conclusions

Our study suggests that a controlled environment with limited capacity for spectators minimizes transmission while allowing resumption of large sporting events during a pandemic.

## Data Availability

The datasets generated and/or analysed during the current study are not publicly available as they contain personally identifiable information as well as identification of individual NCAA teams participating in the 2021 tournament. These datasets, or de-identified versions of them, are available from the corresponding author on reasonable request along with permission from IU Health and/or the NCAA.

## Abbreviations

CDC: U.S. Centers for Disease Control and Prevention
COVID-19: Coronavirus Disease 2019
IU: Indiana University
mRNA: Messenger RNA
NBA: National Basketball Association
NCAA: National Collegiate Athletic Association
NP: Nasopharyngeal
RNA: Ribonucleic acid
RT-PCR: Reverse transcription-polymerase chain reaction
SARS-CoV-2: Severe acute respiratory syndrome coronavirus 2

## References

[1] Domenech-Montoliu S, Pac-Sa MR, Vidal-Utrillas P, Latorre-Poveda M, Del Rio-Gonzalez A, Ferrando-Rubert S, Ferrer-Abad G, Sanchez-Urbano M, Aparisi-Esteve L, Badenes-Marques G (2021). Mass gathering events and COVID-19 transmission in Borriana (Spain): a retrospective cohort study. PLoS ONE.

[2] Menachemi N, Yiannoutsos CT, Dixon BE, Duszynski TJ, Fadel WF, Wools-Kaloustian KK, Unruh Needleman N, Box K, Caine V, Norwood C (2020). Population point prevalence of SARS-CoV-2 infection based on a statewide random sample - Indiana, April 25–29, 2020. MMWR Morb Mortal Wkly Rep.

[3] McCloskey B, Zumla A, Ippolito G, Blumberg L, Arbon P, Cicero A, Endericks T, Lim PL, Borodina M (2020). Mass gathering events and reducing further global spread of COVID-19: a political and public health dilemma. Lancet.

[4] NCAA cancels remaining winter and spring championships [https://www.ncaa.org/about/resources/media-center/news/ncaa-cancels-remaining-winter-and-spring-championships].

[5] Mack CD, DiFiori J, Tai CG, Shiue KY, Grad YH, Anderson DJ, Ho DD, Sims L, LeMay C, Mancell J (2021). SARS-CoV-2 transmission risk among national basketball association players, staff, and vendors exposed to individuals with positive test results after COVID-19 recovery during the 2020 regular and postseason. JAMA Intern Med.

[6] Murray MT, Riggs MA, Engelthaler DM, Johnson C, Watkins S, Longenberger A, Brett-Major DM, Lowe J, Broadhurst MJ, Ladva CN (2020). Mitigating a COVID-19 outbreak among major league baseball players - United States, 2020. MMWR Morb Mortal Wkly Rep.

[7] Moritz S, Gottschick C, Horn J, Popp M, Langer S, Klee B, Purschke O, Gekle M, Ihling A, Zimmermann FDL (2021). The risk of indoor sports and culture events for the transmission of COVID-19. Nat Commun.

[8] COVID-19 Guidance on Multiple Teams in the Same Location [https://www.ncaa.org/sport-science-institute/covid-19-guidance-multiple-teams-same-location].

[9] DiFiori JP, Green G, Meeuwisse W, Putukian M, Solomon GS, Sills A (2021). Return to sport for North American professional sport leagues in the context of COVID-19. Br J Sports Med.

[10] Thompson CN, Hughes S, Ngai S, Baumgartner J, Wang JC, McGibbon E, Devinney K, Luoma E, Bertolino D, Hwang C (2021). Rapid emergence and epidemiologic characteristics of the SARS-CoV-2 B.1.526 Variant - New York City, New York, January 1-April 5, 2021. MMWR Morb Mortal Wkly Rep.

[11] Galloway SE, Paul P, MacCannell DR, Johansson MA, Brooks JT, MacNeil A, Slayton RB, Tong S, Silk BJ, Armstrong GL (2021). Emergence of SARS-CoV-2 B.1.1.7 Lineage - United States, December 29, 2020-January 12, 2021. MMWR Morb Mortal Wkly Rep.

[12] GISAID. Tracking of hCoV-19 Variants. [https://gisaid.org/hcov19-variants/].

[13] Nanduri S, Pilishvili T, Derado G, Soe MM, Dollard P, Wu H, Li Q, Bagchi S, Dubendris H, Link-Gelles R (2021). Effectiveness of Pfizer-BioNTech and Moderna Vaccines in Preventing SARS-CoV-2 Infection Among Nursing Home Residents Before and During Widespread Circulation of the SARS-CoV-2 B.1.617.2 (Delta) Variant - National Healthcare Safety Network, March 1-August 1, 2021. MMWR Morb Mortal Wkly Rep.

[14] Patel DR, Field CJ, Septer KM, Sim DG, Jones MJ, Heinly TA, Vanderford TH, McGraw EA, Sutton TC (2021). Transmission and protection against reinfection in the ferret model with the SARS-CoV-2 USA-WA1/2020 reference isolate. J Virol.

[15] Kim YI, Kim SM, Park SJ, Kim EH, Yu KM, Chang JH, Kim EJ, Casel MAB, Rollon R, Jang SG (2021). Critical role of neutralizing antibody for SARS-CoV-2 reinfection and transmission. Emerg Microbes Infect.

[16] Dan JM, Mateus J, Kato Y, Hastie KM, Yu ED, Faliti CE, Grifoni A, Ramirez SI, Haupt S, Frazier A (2021). Immunological memory to SARS-CoV-2 assessed for up to 8 months after infection. Science.

[17] Bozio CH, Grannis SJ, Naleway AL, Ong TC, Butterfield KA, DeSilva MB, Natarajan K, Yang DH, Rao S, Klein NP (2021). Laboratory-Confirmed COVID-19 Among Adults Hospitalized with COVID-19-Like Illness with Infection-Induced or mRNA Vaccine-Induced SARS-CoV-2 Immunity - Nine States, January-September 2021. MMWR Morb Mortal Wkly Rep.

[18] O'Donoghue AL (2021). Association of University Student Gatherings With Community COVID-19 Infections Before and After the NCAA March Madness Tournament. JAMA Netw Open.

[19] Vest JR, Blackburn J, Cash-Goldwasser S, Peters Bergquist E, Embi PJ (2021). Mask-Wearing Behavior at the 2021 NCAA Men's Basketball Tournament. JAMA.

